# Policy Adjustment in a Dynamic Economic Game

**DOI:** 10.1371/journal.pone.0000103

**Published:** 2006-12-20

**Authors:** Jian Li, Samuel M. McClure, Brooks King-Casas, P. Read Montague

**Affiliations:** 1 Human Neuroimaging Laboratory, Center for Theoretical Neuroscience, Department of Neuroscience, Baylor College of Medicine, Houston, Texas, United States of America; 2 Menninger Department of Psychiatry and Behavioral Sciences, Baylor College of Medicine, Houston, Texas, United States of America; Claremont Graduate University, United States of America

## Abstract

Making sequential decisions to harvest rewards is a notoriously difficult problem. One difficulty is that the real world is not stationary and the reward expected from a contemplated action may depend in complex ways on the history of an animal's choices. Previous functional neuroimaging work combined with principled models has detected brain responses that correlate with computations thought to guide simple learning and action choice. Those works generally employed instrumental conditioning tasks with fixed action-reward contingencies. For real-world learning problems, the history of reward-harvesting choices can change the likelihood of rewards collected by the same choices in the near-term future. We used functional MRI to probe brain and behavioral responses in a continuous decision-making task where reward contingency is a function of both a subject's immediate choice and his choice history. In these more complex tasks, we demonstrated that a simple actor-critic model can account for both the subjects' behavioral and brain responses, and identified a reward prediction error signal in ventral striatal structures active during these non-stationary decision tasks. However, a sudden introduction of new reward structures engages more complex control circuitry in the prefrontal cortex (inferior frontal gyrus and anterior insula) and is not captured by a simple actor-critic model. Taken together, these results extend our knowledge of reward-learning signals into more complex, history-dependent choice tasks. They also highlight the important interplay between striatum and prefrontal cortex as decision-makers respond to the strategic demands imposed by non-stationary reward environments more reminiscent of real-world tasks.

## Introduction

Knowing how to behave adaptively reduces, in most circumstances, to knowing the consequences of available actions, or, how much reward each action will garner on average. Practically speaking, this is a daunting problem. This is particularly so since the reward associated with different actions depends on a wide variety of factors such as one's history of actions, the behavior of competitors, and even stochastic changes in the environment through time. For example, a bee's decision to harvest nectar from one flower has the inevitable consequence of decreasing the returns from that flower and increasing the returns from non-sampled flowers (the nectar levels can recover). In foraging theory, if the amount of available prey (i.e. reward) is greater than the appetite of the predators then the food supply will increase [1]. Sudden unexpected shocks can also have significant effects on the costs and benefits associated with different actions. In general, the reward available in the future depends in complex ways on a possibly overwhelming variety of environmental factors [2]–[3].

Despite this apparent complexity of action-reward relationships presented by the world, most work in neuroscience and psychology has focused on fixed action-reward dependencies and studied the change of action-reward contingencies in block design paradigms such as Wisconsin Card-sorting task (WCST), reversal learning paradigms and extinction paradigm where in certain block of trials the action-reward contingency is fixed [4]–[6]. This trend persists in recent neuroimaging studies in humans, in which operant learning paradigms have been studied extensively [7–16, but see 17]. These studies suggested that brain areas associated with the mesolimbic dopamine system (i.e. striatal structures, prefrontal cortex) play an important role in reward learning and action selection [18]–[21].

In this paper, we study changes in action selection reflective of changes in reward expectation in a series of tasks in which earned reward depends in complex ways on previous actions. The rationale of this study was to fit each subject's behavior through time with a continuous error-based learning model (e.g. actor-critic model) [5], [9]–[14] to predict subject's consequent action selection and correlated brain activity in a series of tasks where reward contingency is a function of both subject's immediate choice and choice history. We then asked how subject responded to unexpectedly introduced new reward structures since subjects are required to develop different strategies for these reward structures and usually the adjustment of strategies correspond to increasingly exploratory actions by the subjects; hence they would give us opportunities to observe the interplay between control signals in striatum and prefrontal cortex during the switches of periods when action-reward dependencies vary slowly through time and periods where subject's behavior becomes more variable and depends less on previous experience.

## Results

The experimental design and subjects' performance in these tasks has been described previously [9], [22]–[23]. We reviewed subjects' behavioral tendencies, and note that all of the subjects performed in accord with these summaries on each task individually, and switched behavioral strategies rapidly when the tasks were switched in our current manipulation.

### MS→RO task (Matching Shoulder→Rising Optimum)

**Figure 1:**
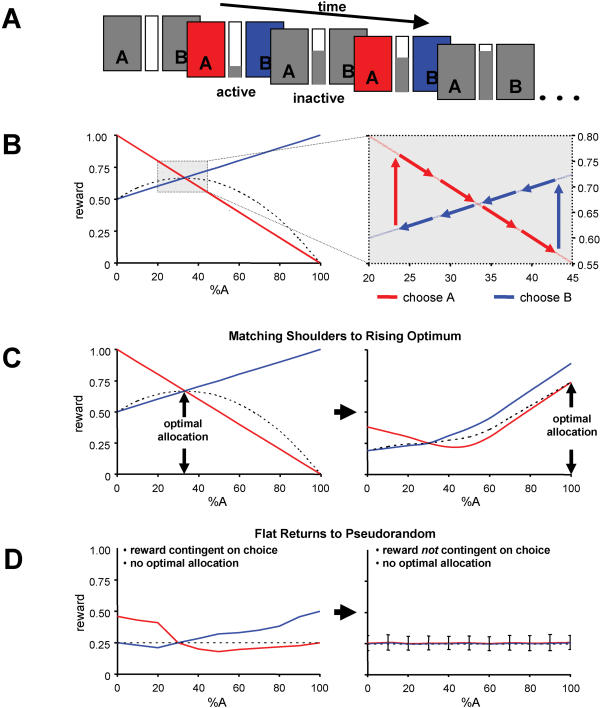
Experimental design. **(A)** Subjects were engaged in two decision-making tasks in which they were instructed to choose from one of two actions (A or B) with the goal of obtaining and maintaining maximum earned reward as indicated by a centrally located reward bar. **(B)** Example of reward structure: Central bar height (reward) depends on two variables: 1) current decision (A or B, Red and Blue trace correspondingly) and 2) the percentage of choice A (%A) made over the past 20 trials (x-axis). The initial %A is set to be 50%. In cases in which participants chose choice A in *more* than 33% of the previous 20 trials, a B choice results in greater reward than an A choice. In cases in which participants chose choice A in *less* than 33% of the previous 20 trials, an A choice results in greater reward than a B choice. Thus, to the right of the crossing-point of the two reward functions, ‘B’ choices both increase reward and move subjects to left on the x-axis. However, as subjects move left past the crossing-point, A choices begin to yield greater reward than B choices and move subjects right on the x-axis. In this example, the crossing-point represents the optimal allocation to A, as the average return at all other allocations is less than at the crossing-point of the two reward structure (indicated by the dashed line). **(C)** In the first task, participants made 125 decisions with reward determined by the matching shoulders (MS) structure (left panel), followed by 125 decisions with reward given by the rising optimum (RO) structure (right panel). **(D)** In a second task, participants began with the flat returns (FR) structure (left panel) and switched to a pseudorandom (PR) task (right panel). In the FR task, all choice strategies yield the same average return (dashed line). In the PR condition, reward was randomly determined independent of choice but was set to give the same mean and variance of rewards as was earned in the FR structure.

In both the matching shoulders and rising optimum tasks, subjects have a strong tendency to perform near the crossing point in the reward functions (see Figure 1B legend for detail). This can be understood by considering how earned reward changes near the crossing point. Assuming that subjects choose A at the crossing point, the percent allocation to A (%A) will increase, resulting in decreased subsequent earned reward for selecting A (Figure 1B). Reward can be increased by switching to choice B, which also decreases the percent allocation to A, returning subjects to the crossing point. The converse sequence of events occurs if B is initially selected. As long as subjects tend to select in accord with which choice is expected to produce the greatest immediate reward (Herrnstein called this melioration, [3], [24]), then they will perform at the crossing point in the reward functions (∼33% allocation to A) in both the MS and RO tasks.

**Figure 2:**
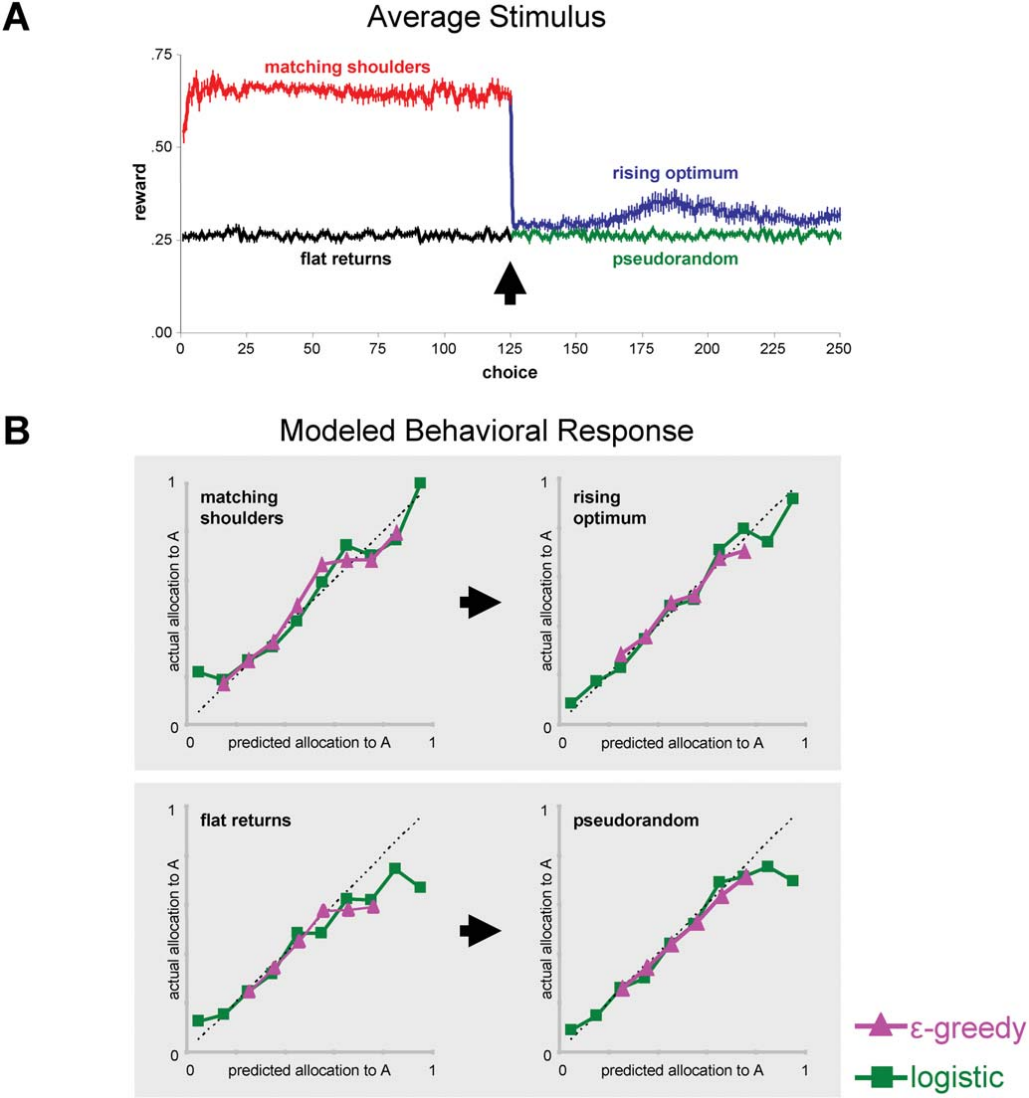
Experienced rewards and modeled behavioral response. **(A)** The switch from matching shoulders (MS) to rising optimum (RO) reward structures was signaled by a large decrease in immediate reward return (∼60%). However, the switch from the flat returns (FR) structure to the pseudorandom (PR) condition did not elicit a similar change in experienced reward. Reward S.E. is indicated by vertical bars at each choice. **(B)** Subject decisions were predicted using a reinforcement learning model with two different methods to determine the probability to choose a certain action (ε-greedy method and sigmoid method). For both methods, we assume that subjects maintained independent estimates of the reward expected for each choice, A and B, and updated these values based on experienced rewards using the Rescorla-Wagner learning algorithm. Choices were assumed to be 1) probabilistically related to choice values according to a sigmoid function (softmax method, green curve) or 2)have a fixed probability of 1-ε/2 for choice associated with bigger weight (ε-greedy method, pink curve). Panel B indicates the relationships between predicted and actual choices. Decisions were binned (x-axis) based on predicted likelihood that subjects would choose A. Y-values indicate the actual average allocation to A for all choices within each bin. Linear regression shows there is a strong correlation between predicted and actual choices. (MS: r = 0.97, RO: r = 0.99, FR: r = 0.97, PR: r = 0.97 for softmax method; MS: r = 0.97, RO: r = 0.99, FR: r = 0.95, PR: r = 0.99 for ε-greedy method). ε-greedy method restrict each subject only have one probability to choose certain choice (A or B) over 250 trials, while sigmoid method allows subject to generate different probability to choose specific choice for each trial in the 250 trial task. This is why ε-greedy method does not cover all the spectrum of X-axis as sigmoid method does.

In the MS task, performing at the crossing point is the optimal solution [9]. However, in the RO task it is grossly sub-optimal. If subjects were to select button A on every choice in the RO task, they would experience a temporary decrease in earned reward that would subsequently reverse to produce the maximum average return (Figure 1B). This optimal policy (selecting A only) is an unstable equilibrium point in subjects' action selection policy due to the fact that at high allocation to A, choices to B produce greater immediate reward (Figure 1C). In the MS→RO task, subjects show evidence for both behavioral equilibria after the reward structure switch. They began performing near the crossing point, showing a temporary excursion to greater allocation to A (%A), and then reverted again to performing near the crossing point (Figure 2A).

### FR→PR task (Flat return → Pseudorandom)

Regardless of the pattern of choices in the FR and PR tasks, the average earned reward will be the same (Figure 1D, dot line; Figure 2A). As in MS and RO, the FR reward structure possesses a crossing point in the reward functions that acts as a stable selection strategy (Figure 1D).

Subjects always performed the PR task immediately after the FR task. Furthermore, we configured the reward returns in PR so that the mean and variance in rewards that subjects experienced in PR task were equal to what the subjects earned on the FR task (Figure 1D). Unlike the FR task, reward in the PR paradigm was randomly determined and was not dependent on the subjects' choices (randomly drawn from a uniform distribution). Under these conditions (PR), subjects tend to perform randomly, evenly distributing their choices between A and B. The transition from performing near the crossing point in the FR task (∼40% allocation to A) to equally distributing choice in the PR task (∼50% allocation to A) occurs at variable delays across subjects (see below for discussion).

### Reinforcement learning model of reward learning

Reward learning requires monitoring the expected reward for the available actions (A and B), and biasing choices in favor of the action with highest expected reward. We modeled this process using the two reinforcement learning models described above (Figure 2B). The sigmoid model assumes that subjects tend to select the choice associated with the greatest model weight (i.e. more likely to selected A when *w_A_*>*w_B_*). Furthermore, the probability that subjects select the choice with the greater estimated weight is expected to scale with the difference in weights (*w_A_–w_B_*), while the ε-greedy method assumes a probability of 1-ε/2 to the choice with bigger weight (w*). To test these predictions, we calculated *w_A_–w_B_* at the time of every choice and arranged choices in order of increasing weight difference for the sigmoid action selection method. We then compared the observed probability of selecting A (*P_A_*) by subjects with the probability predicted by the logistic decision function (Green, Figure 2B). For all 4 reward structures, this analysis revealed a strong correlation between observed and estimated probabilities of selecting choice A (MS: r = 0.97, RO: r = 0.99, FR: r = 0.97, PR: r = 0.97). For the ε-greedy method, we assigned individual subject's probability to the choice associated with bigger weight to be 1-ε/2 and probability for the other choice is thus ε/2. We then arranged choices in order of increasing probability of choosing a specific choice (A or B) and then compared the observed probability of selecting A (*P_A_*) by subjects with that predicted by the ε-greedy decision function (Pink, Figure 2B). This analysis revealed a similar fitting as the softmax action selection method (MS: r = 0.97, RO: r = 0.99, FR: r = 0.95, PR: r = 0.99) both in behavioral fitting and further neural correlates mapping.

### Neural correlates of prediction error

**Figure 3:**
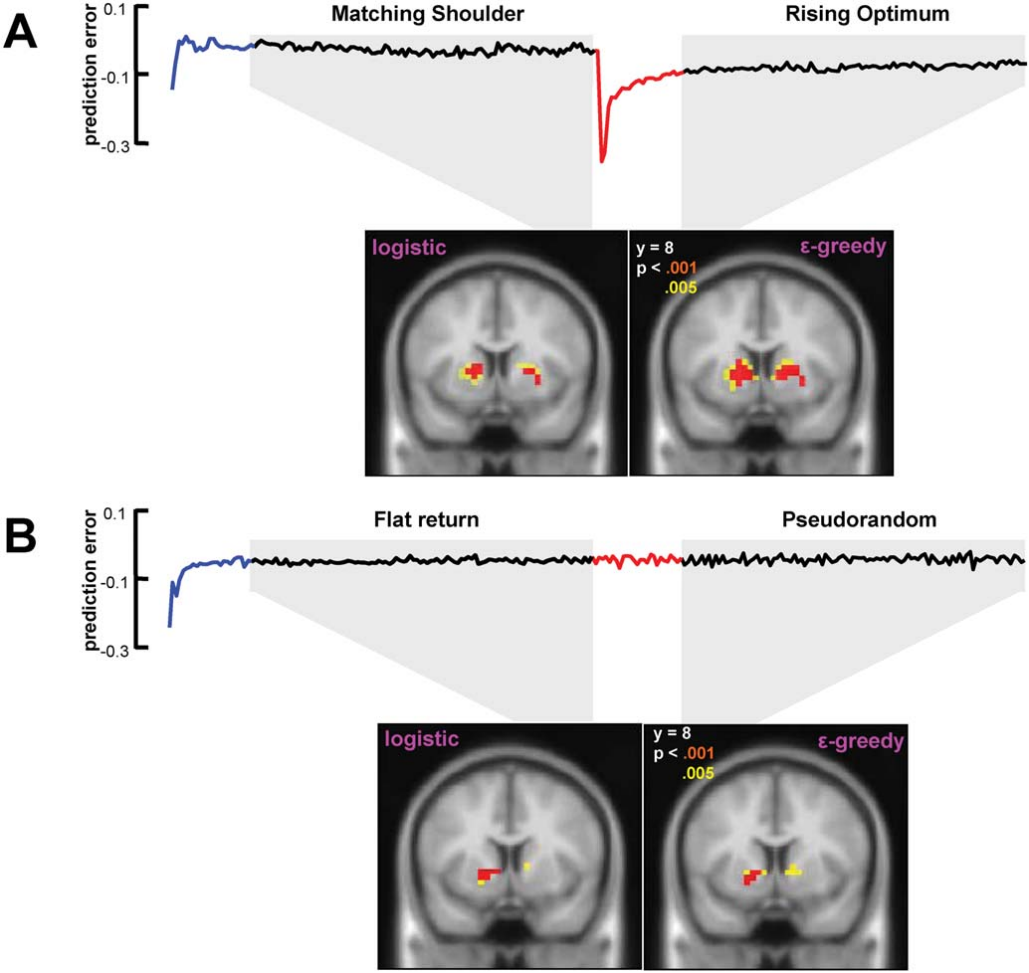
Activity in ventral striatum correlated to prediction errors with two different models. For each choice, a prediction error was generated by comparing the reward experienced by the subject with the current modeled weight value for the choice. Traces in figure 3 indicate the average prediction error, δ*(t)*, across subjects for two different tasks. Neural activity corresponding to prediction errors that were generated from independently fitting reinforcement model to our 2 different tasks (MS → RO and FR → PR) using two different methods (softmax and ε-greey) was identified though general linear model (GLM) analyses. Peak activities for left and right striatum are located at MNI [−12, 8, 8] and [24, 12, −4] for MS→RO task and [−16, 8, −4] and [12, 8, 0] for FR→PR task using softmax method. Activity in ventral striatum correlates with the magnitude of prediction errors in the exploitation periods of both tasks (red: p<0.001; yellow: p<0.005, uncorrected).

Reinforcement learning model states that learning signals (prediction error) are used to update and monitor the value of choices. In our experiment, we used prediction errors estimated from the model and applied it as one of the regressors in a general linear model (GLM) to imaging data. We find that the prediction error signal estimated from two methods (softmax action selection and ε-greedy) correlates with activity in the ventral striatum in both of our tasks with different reward structures (Figure 3).

### Correlation with reward prediction error

The reinforcement learning model fits to the behavioral data provide estimates of the reward prediction error experienced after every choice. These prediction errors were used to produce regressors that were further fit to subjects' functional imaging data.

In the FR→PR task, the BOLD signal in regions of the ventral putamen correlated significantly with estimated prediction error signals (p<0.005, uncorrected) using both methods (softmax and ε-greedy). However, at a threshold of p<0.005 (uncorrected), we find that no areas other than visual cortex are significantly correlated with estimated prediction error signals in the matching shoulders to rising optimum (MS→RO) task. We reasoned that the lack of correlation with estimated prediction error in this task may result from the fact that the large negative prediction error and prolonged recovery phase produced by the change in reward paradigm (Figure 2A, Figure 3, blue and red traces) may dominate the overall fitting. To test this, we excluded the period of time encompassing the first 25 choices following the onset and switch in reward structures from the analysis. With this correction, BOLD signals in ventral putamen correlated significantly with estimated prediction error signals from both methods (p<0.001, uncorrected). This result suggests BOLD signals in ventral striatum (putamen) can be predicted by prediction errors (PE) when action-reward dependencies vary slowly through time where PE fluctuates around 0 (Table 1, Figure 3), but not in phases where subject's behavior becomes more variable and is less dependent on previous experience.

**Table 1:**
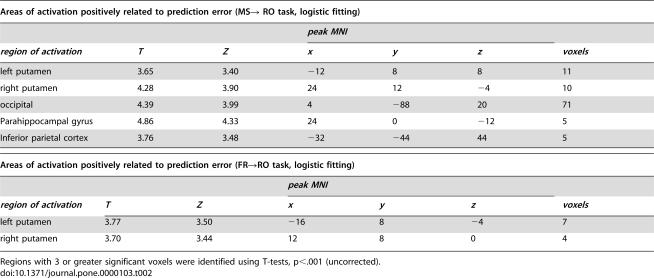


Areas of activation positively related to prediction error (MS→ RO task, logistic fitting)						
			*peak MNI*			
*region of activation*	*T*	*Z*	*x*	*y*	*z*	*voxels*
left putamen	3.65	3.40	−12	8	8	11
right putamen	4.28	3.90	24	12	−4	10
occipital	4.39	3.99	4	−88	20	71
Parahippocampal gyrus	4.86	4.33	24	0	−12	5
Inferior parietal cortex	3.76	3.48	−32	−44	44	5

These results are further confirmed by fitting our learning model to each of the 4 sub-tasks (MS, RO, FR, and PR) independently. Prediction errors generated in this manner, omitting periods immediate after the introduction of new reward structures (25 trials, Figure S3, red and blue), correlate with BOLD signals in the same area of the ventral striatum in each of the four sub-tasks (Figure S3). This indicates that, when behavior is relatively stable, the ventral striatum is engaged to dynamically track ongoing reward estimation errors. Overall, these results correspond well to a recent report that prediction error-like signals occur in the striatum in an operant learning paradigm [12].

### Brain activity during periods of unexpected reward structure switches

We hypothesized that the unexpected salient events can possibly act to indicate possible changes in reward contingency or reward paradigm and they may trigger further exploratory behaviors by subjects. There were three time points in the behavioral tasks that reliably signaled significant changes in reward paradigms which can also be confirmed from deviated prediction error signals (Figure 3). We consider brain areas that are activated in all of these instances as involved in abstract rules monitoring and detection and their activities can be triggered by salient events (dramatic immediate reward change in our case, [25]–[26]) and further help to promote more exploratory behaviors by subjects in order to determine more optimal strategies for current reward structure. We identified these areas using a conjunction analysis (intersection of areas significantly correlated at p<0.001, uncorrected for multiple comparisons, in each instance, [27]). The three points include the beginning of both tasks (first choices in MS and first choices in FR), and the instant when the task paradigm switched from MS to RO. In the MS→RO task, subjects invariably played near the crossing point in the MS task (Figure S1), which resulted in a significant (60%) drop in earned reward when the task switched to RO reward structure. This change caused subjects to alter (even if briefly) behavioral strategy (Figure S2).

**Figure 4:**
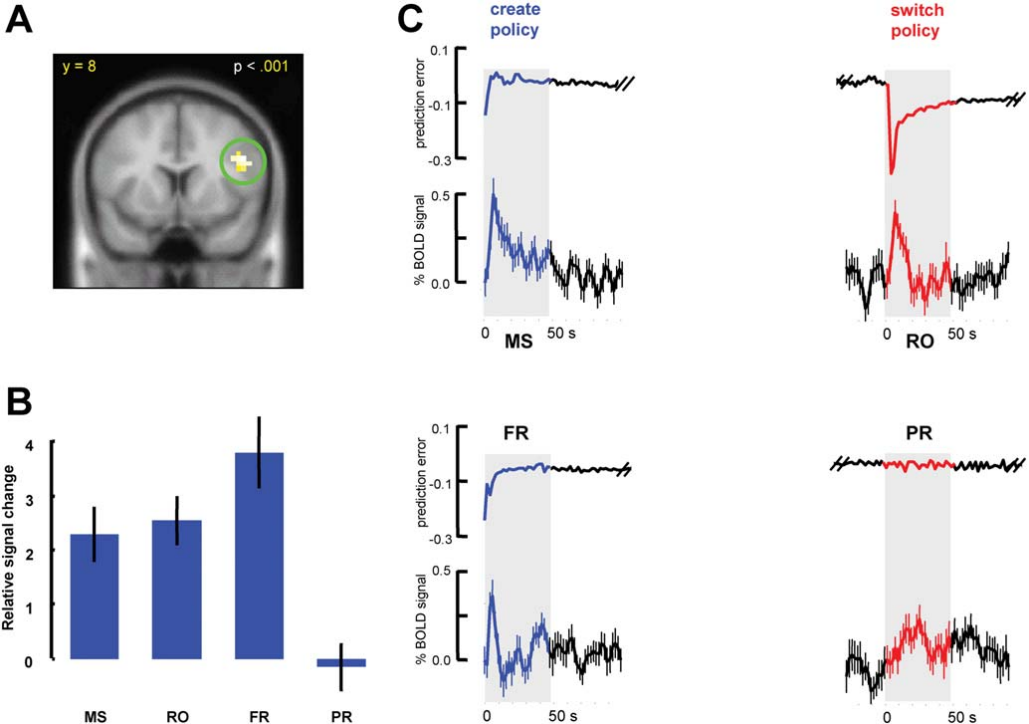
Inferior frontal gyrus (IFG) and anterior insula respond to reward structure switches. (A)The IFG activity was identified by a conjunction analysis as brain responds to the introduction of new reward structures. (B) Relative BOLD signal changes of IFG in four different sub-tasks are estimated by general linear model (GLM). Activities at the onsets of reward structure MS, RO, FR are significantly bigger than zero (p<0.001, one sample t-test). (C) BOLD signal changes were further confirmed by a region of interest (ROI) analysis in the four sub-tasks. These periods correspond to initially learning the reward structure of the tasks (blue) and adapting to changes in reward structure in the middle of the MS→RO task (red). Qualitatively equivalent results were also obtained for the anterior insula.

Three brain areas were identified by the conjunction analysis including the bilateral anterior insula and a region in the inferior frontal gyrus (IFG) (See Table 2 for detailed description). The region identified in the insula has been implicated in responding to cognitive conflict and behavior inhibition [21], [28]. The other region we identified lies on the IFG and extends into the frontal operculum (BA 44, Figure 4). This region has been identified under conditions requiring increased attention and changes in behavior [29].

**Table 2:**
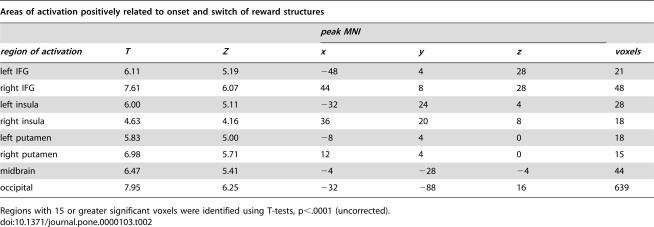


Areas of activation positively related to onset and switch of reward structures						
			*peak MNI*			
*region of activation*	*T*	*Z*	*x*	*y*	*z*	*voxels*
left IFG	6.11	5.19	−48	4	28	21
right IFG	7.61	6.07	44	8	28	48
left insula	6.00	5.11	−32	24	4	28
right insula	4.63	4.16	36	20	8	18
left putamen	5.83	5.00	−8	4	0	18
right putamen	6.98	5.71	12	4	0	15
midbrain	6.47	5.41	−4	−28	−4	44
occipital	7.95	6.25	−32	−88	16	639

Regions with 15 or greater significant voxels were identified using T-tests, p<.0001 (uncorrected).

Another time point in our experiment that might be associated with exploration is the task switch in the FR→PR paradigm. Subjects changed from performing near the crossing point (∼40% A%) in the FR task to random selecting behavior in the PR task (∼50% A%) (Figure S2). There are two differences between the task switch in FR→PR and MS→RO that underlie why we did not include it in the conjunction analysis. First, in FR→PR, subjects switch to a null behavioral strategy (random play). Thus it is unclear to what degree subjects are exploring new behavioral strategies as opposed to simply omitting strategic play. Secondly, the reward structure switch in FR→PR task does not produce as dramatic a signal of changed reward contingencies as in the MS→RO paradigm. In MS→RO task, the bar height decreases approximately 60% at the task switch. For FR→PR task, the mean and variance in reward were unchanged after the reward structure switch. Subjects may therefore require more time to discover and respond to the change in reward contingencies and the time for each subject to discover and respond could vary across individuals. This is further confirmed by the fact that reward prediction error signal estimated from both of our models does not indicate any significant changes during the switch from FR to PR reward structure (Figure 3). Consistent with this, activity does increase in the area of PFC identified by the conjunction analysis following the switch in FR→PR. However, the amplitude is reduced and the duration of activity is prolonged relative to MS→RO task. This is the expected outcome were subjects to discover and respond to the task switch at more delayed and variable times.

## Discussion

Using a continuous decision-making task with four different dynamic reward structures underlying subjects' action-outcome contingencies, we found in this paper that differential involvement of brain areas in action selection and learning during different time periods of the task. Two model-based regression analyses showed BOLD activities in the ventral striatal structure correlate with prediction error signal. However, a sudden introduction of new reward structures engages more complex control circuitry in the prefrontal cortex (inferior frontal gyrus and anterior insula) and is not captured by a simple actor-critic model. Separately, these two systems have been the subject of numerous investigations of decision-making. The first of these systems, the ventral striatum, is believed to be involved specifically in maintaining and updating the expected reward value of actions. This is accomplished through interactions with the mesolimbic dopamine system which activates in accord with ongoing reward prediction error signals [18]. In the striatum, dopamine is known to modulate synaptic plasticity [19] allowing for the activity of these neurons to encode action value [30]. Functional MRI studies have demonstrated that the striatum is clearly involved in biasing action selection in accord with current action values [13], and that activity in this structure changes in accord with ongoing prediction errors [10], [31]–[32]. Our findings show that these results hold during periods of conditional action-selection learning in each of the four different reward structures when, presumably, the striatum is the primary determinant of behavior. Our results distinguished from previous studies by using different underlying reward functions originally derived from Herrnstein's matching law [3], [22], [24] that reward associated with each choice depends not only on the current choice but also subject's previous choice history, while most of the previous studies adopted fixed action-reward contingencies paradigms [11]–[14], [33]. Animals have to face a non-stationary world and the amount of reward expected from a contemplated action depends in complex ways on the history of an animal's choices. This can dramatically change the likelihood of rewards collected by the same choices in the near-term future, and our result indicates similar neural correlates are required in reward-learning tasks more reminiscent of real-world environment.

During periods where immediate reward return fluctuates dramatically (at the beginnings of both tasks, and in the middle of MS->RO task), activity in the striatum is not well captured by reinforcement learning models, suggesting that the striatum is not strongly involved in action selection during these periods. In these periods, brain activity is increased in two areas of the prefrontal cortex: in the inferior frontal gyrus as well as bilaterally in the anterior insula. The insula has primarily been implicated as responding to disgust, pain, and other aversive stimuli [34]–[36]. In terms of decision-making tasks such as ours, the insula has also been found to be activated by changes in reward contingencies [21]). Together, these findings have been taken to suggest that the anterior insula (as well as part of the lateral orbitofrontal cortex) is also involved in inhibiting old action-selection patterns. The IFG, by contrast, is much more strongly linked to maintaining and switching between action strategies [37]. As an example, a recent study by Schmitz and colleagues [29] investigated which brain systems are involved when subjects are required to lift varying weights. When the required motor plans were regular (either because the weight was constant or alternated regularly between two values), the level of activity observed in the IFG was significantly less than when the motor plan had to be constantly reformulated (random sequence of weight change). Another study by Cools et al. [38] demonstrated that BOLD signal change in the lateral prefrontal cortex (PFC) was observed during the lower-order switching between concrete objects and higher-order switching between abstract task rules. We interpret our observed IFG activity accordingly: during periods when higher order action strategies are in greater flux, increased activity results in the IFG. The overall task requirements, to maximize earned reward and to select from two constant actions, were unchanged. Given this, it is perhaps not surprising that we find activity changes restricted to posterior prefrontal cortex. Recent experiments have led to the hypothesis that there may be a rostro-caudal hierarchical organization in lateral prefrontal cortex such that higher-order task goals are maintained in more anterior aspects of PFC [20]. We hypothesize that the IFG is specifically involved in maintaining and changing between action strategies within a set of task requirements.

### Involvement of both frontal and striatal systems in decision-making under uncertainty

Numerous recent reports have identified the striatum and prefrontal cortex as two parallel, and often times competing, systems that interact to guide behavior [25]–[26], [33], [35], [39]–[42]. The current prevalence of studies highlighting the competition between these two systems is probably more a reflection of what constitutes a hallmark problem in economics and philosophy than the general nature of interaction of these two systems [43]. Daw and colleagues [33] discussed a possible complimentary interaction between the striatum and prefrontal cortex in which control of behavior is given to the system which is best suited to the current problem. Other hypotheses suggest that dopaminergic signals from ventral tegmental area (VTA) serve as a “gating” mechanism to the prefrontal cortex (PFC) to update goal representation and change action selection policy [25–26, 41, 44–45). Interestingly, a recent paper by Pasupathy and Miller [42] showed that in a conditional visual-motor learning task, rapid changes of striatum activity as well as a slower trend changes in the prefrontal cortex have been observed. The authors interpreted their result to support both hypotheses. In our task, the co-occurrence of PFC activity and dramatic changes in prediction error signals (Figure. 4) suggest that learning in the prefrontal cortex might be triggered by midbrain dopaminergic signals as the dopamine-gating hypothesis indicates [25]–[26]. The positive BOLD signals also detected at brain areas such as visual cortex, midbrain (locus ceruleus) and striatum (Table 2) during the introduction of MS, RO and FR tasks imply multiple brain mechanisms, which are responsible for novelty or salience detection, arousal increasing and attention direction [46]–[47], might also be recruited to detect and further respond to the reward structure changes.

### Involvement of frontostriatal circuitry in exploration-exploitation tradeoff

The exploration-exploitation dilemma necessarily arises when decisions are made without complete knowledge of the world. Choices can be made that deliver the maximum reward based on what is currently known (*exploitation*) or to try unknown alternatives in the hope of discovering better actions, a strategy known as *exploration*. Exploration has the potential to greatly improve performance as it allows for the discovery of optimal actions. However, it can also be very costly. If the optimal strategy were already known, then exploration will only serve to reduce rewards. Any decision-maker will have to face the exploration-exploitation balance dilemma. Animals need to keep a stable behavioral strategy (exploitation) while in the meantime maintaining the flexibility to adapt to new environment once enough salient evidence has accumulated indicating previous strategy is no longer optimal. In our task, we specifically avoided studying how exploration and exploitation trade off as behavioral strategies in this experiment (but see Yu and Dayan [48], [31], [49]). Instead, we hypothesize that control of behavior switches between the striatum and prefrontal cortex as the demands for these two behavioral strategies change. In our task, we manipulated the experimental design to create choice situations that reliably 1) elicit exploitation responses and 2) demonstrate reward paradigm change and further demand more explorative behaviors in a continuous decision making paradigm. This allowed us to directly target those brain areas involved separately in exploration and exploitation. As a result, our data might suggest that different weightings of striatum-prefrontal cortex circuits may dominate people's strategy selection.

These findings should open the door to investigations of how prefrontal and striatal systems function together to direct people's actions during decision-making under uncertainty.

## Materials and Methods

A total of 46 subjects were recruited for this study (22 male). All subjects were right hand dominant and were on average 32 years old (S.D.±9.3). Subjects had no history of psychiatric illness. Informed consent was obtained using a consent form approved by the Baylor College of Medicine Institutional Review Board.

Scanning was performed in a head-dedicated Siemens Allegra scanner with field strength 3T. Prior to the experiment, high-resolution T1-weight anatomical images (1 mm×1 mm in-plane resolution) were acquired to allow localization of functional activity. Whole brain echo-planar images (EPI) were acquired in 26 axial slices (3.4×3.4×4 mm width) parallel to the AC-PC line. Images were acquired with a repetition time (TR) of 2s, an echo time (TE) of 40ms, and flip angle of 90°.

### Experimental Task

Subjects lay supine with their head in the scanner bore and observed the rear-projected computer screen via a 45° mirror mounted to the head coil. Choices were registered using two MRI-compatible button boxes. Selections to A were made by pressing any button with the left hand and selections to B by pressing any button with the right hand for half number of the subjects and in the reversed pattern for the other half. After each selection, the central reward bar obtained a new height dependent on earned reward. Following this, the buttons (A and B) on the screen were disabled and turned gray for 1.25 s. Subjects were instructed that they could not make further selection until the buttons on the screen turned back to normal color from gray.

Subjects engaged in two repeated play, two-alternative decision-making tasks in which they were instructed to choose from one of two actions (A or B) with the goal of obtaining and maintaining maximum earned reward (Figure 1A; [8], [23]). The central bar height (reward) is controlled by two variables: 1) Current choice made (A or B), if the subject chooses A, then the reward received will be along the red line, otherwise, the reward will be on the blue line; 2) Subject' choice history: the percentage of choice “A” (%A) selected in the past 20 choices. The initial %A value is set to be 50%. As task proceeds, the %A is updated (a 20-choice moving window) as a result of each choice (A or B) subjects made. The tasks were modified for use in fMRI by pacing the rate at which choices are made to no faster than one every 1.25 s. The mean reaction time was slightly less than 2 s. Each task required subject to make 250 selections. After the first 125 selections, the reward structure was switched (Figure 1B, C). Subjects were not instructed that these switches would occur. In the first task, the reward structure was initially defined by the matching shoulders (MS) paradigm and was then switched to the rising optimum (RO) reward paradigm (MS→RO task; Figure. 1*C*). The other task began with the flat returns (FR) paradigm and was switched to pseudo-random (PR) returns at the switch (FR→PR task; Figure. 1*D*). In all reward paradigms except pseudo-random, earned reward depended on two variables: (1) the subject's choice (A or B; corresponding to red and blue reward curves in Figure 1C, D, respectively), and (2) the percent of the last 20 choices made to choice A (%A, allocation to A; x-axis on plots in Figure. 1B, C, D). Allocation to A was set to 50% at the beginning of both tasks.

### Data Analysis

Imaging data was analyzed using SPM2 [50] and xjView (http://people.hnl.bcm.tmc.edu/cuixu/xjView/). Functional images were realigned, corrected for slice timing, coregistered with a canonical brain in MNI coordinates, resliced to 4x4x4mm and smoothed with an 8mm FWHM Gaussian kernel prior to analysis.

The prediction error signal, δ*(t)*, determined by fitting the behavioral data, was used to produce a regressor through convolution with a canonical hemodynamic response kernel. To find brain voxels sensitive to changes in reward paradigm, we used a regressor with a single hemodynamic response function offset to the time of reward structure switch (beginning of each reward structure).

Regressors were fit independently to data from each voxel in the functional brain images using standard linear model methods. A random effects analysis was conducted by performing one-sample t-tests over best fitting beta amplitudes produced by linear model fitting. Brain areas are considered significantly activated that are composed of at least 5 contiguous voxels significant at *p*<0.005 with peak significance in the cluster of at least *p*<0.001.

### Behavior Fitting–Modeling

Subjects' decision-making was modeled with a reinforcement learning algorithm. We assume that subjects maintained independent estimates of the reward expected for each choice, A and B, and updated these values based on experienced rewards. In particular, we assume choice values (*w_A_* and *w_B_*) were updated according to a Rescorla-Wagner learning algorithm.

We used two methods to assign probabilities to each choice: 1) logistic method and 2) ε-greedy method. In logistic method, choices were assumed to be probabilistically related to choice values according to a sigmoid function with slope *m*:
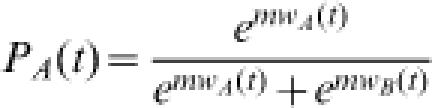



While in ε-greedy method, one of the two alternative choices will be assigned with probability 1-ε/2 if the weight associated with that choice is bigger than the other and the probability of choosing the other choice is thus ε/2. When the weights associated with two choices are equal, then one choice will be randomly assigned with probability 1-ε/2, the other ε/2.
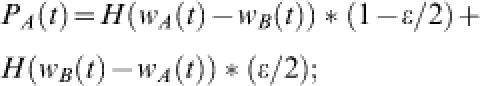

*Where H(x) is a Heaviside step function and defined by*

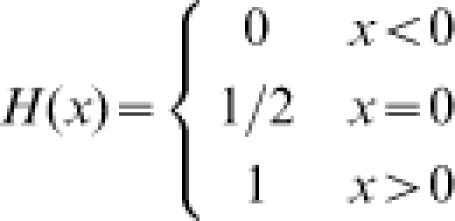



In both methods, for each choice (denote the choice by *), the reward experienced by the subject *r(t)* was compared with the current modeled weight value W*(t) to produce a prediction error δ*(t)*:
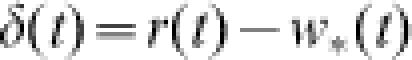



The prediction error served as a learning signal that was used by both methods to improve modeled action weights by an amount governed by the learning rate*λ*:
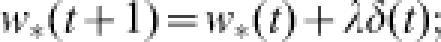



The quality of both model fittings was determined by how well they are able to account for the actual pattern of subjects' choices. This was quantified as the product of the modeled probabilities of all of the subjects' choices, the log likelihood estimate (LLE) (i.e. log Π_t_ P*(t)) in two models. Free parameters in sigmoid method included the learning rate (λ) and the slope of the sigmoid decision function (*m*), while another two free parameters: the learning rate (λ) and greedy factor (ε) are used in ε-greedy method. Initial weights for both methods were held such that *w_A_* = *w_B_* = 0.5. The models were fit so as to maximize LLE for each subject individually using a simplex method. To avoid local minima in parameter fitting, fitting was initiated from 20 randomly determined starting values and the best fit was taken across all final parameter values. The learning rate was restricted to values between 0 and 1; the sigmoid slope was restricted to positive values and the greedy parameter (ε) was restricted to values between 0 and 1.

## Supporting Information

Figure S1 Individual subject performance variability in both tasks. These four panels represent how individual subject averagely perform in 4 different reward structures (MS, RO, FR, PR). Immediate reward subject receive from each choice they make depends on two variables: 1) current decision (A or B, Red and Blue trace correspondingly) and 2) the percentage of choice A (%A) made over the past 20 trials (x-axis). Each subject's average behavior is represented by a triangle on each reward structure plot. Most subject perform around the optimal strategy (cross point of red and blue curve) in the MS task, while in RO task, subjects tend to split along the %A and many subject were restricted to the crossing point which is not the optimal strategy anymore. In FR task, subjects were still slightly attracted by the crossing point while in the PR task subjects were randomly distributed around the 50% %A point.
(1.44 MB TIF)

Figure S2 Various behavioral responses subjects performed in both tasks. Subjects quickly adjusted to the optimal strategy at the beginning of both tasks (MS and FR). The switch from matching shoulders (MS) to rising optimum (RO) reward structures was signaled by a large decrease in immediate reward return (Fig. 2) and could possibly trigger the more exploratory behavior in the RO task. However, the switch from the flat returns (FR) structure to the pseudorandom (PR) condition did not elicit a similar change in experienced reward and thus although the general behavioral patterns in FR and PR task are different (∼40% %A in FR task and ∼%50 %A in PR task), there is no evidence indicating a reliable exploratory phase in PR task. %A S.E. is indicated by vertical bars at each choice.
(1.60 MB TIF)

Figure S3 Ventral striatum as the neural correlate of average prediction error, δ(t), across subjects for four different sub-tasks using softmax method. Neural activity corresponding to prediction errors generated from independently fitting the softmax reinforcement model to 4 sub-tasks (MS, RO, FR, PR). Activity in ventral striatum correlates with the magnitude of prediction error in each sub-task (MS, RO, FR, PR) (red: p<0.001; yellow: p<0.005, uncorrected) when reward contingencies vary slowly through time.
(3.82 MB TIF)
