# Supplementary figures and images for: Policy Adjustment in a Dynamic Economic Game
Source: PLoS One. 2006 Dec 20;1(1):e103. doi: 10.1371/journal.pone.0000103 (PMC1762366; doi:10.1371/journal.pone.0000103)

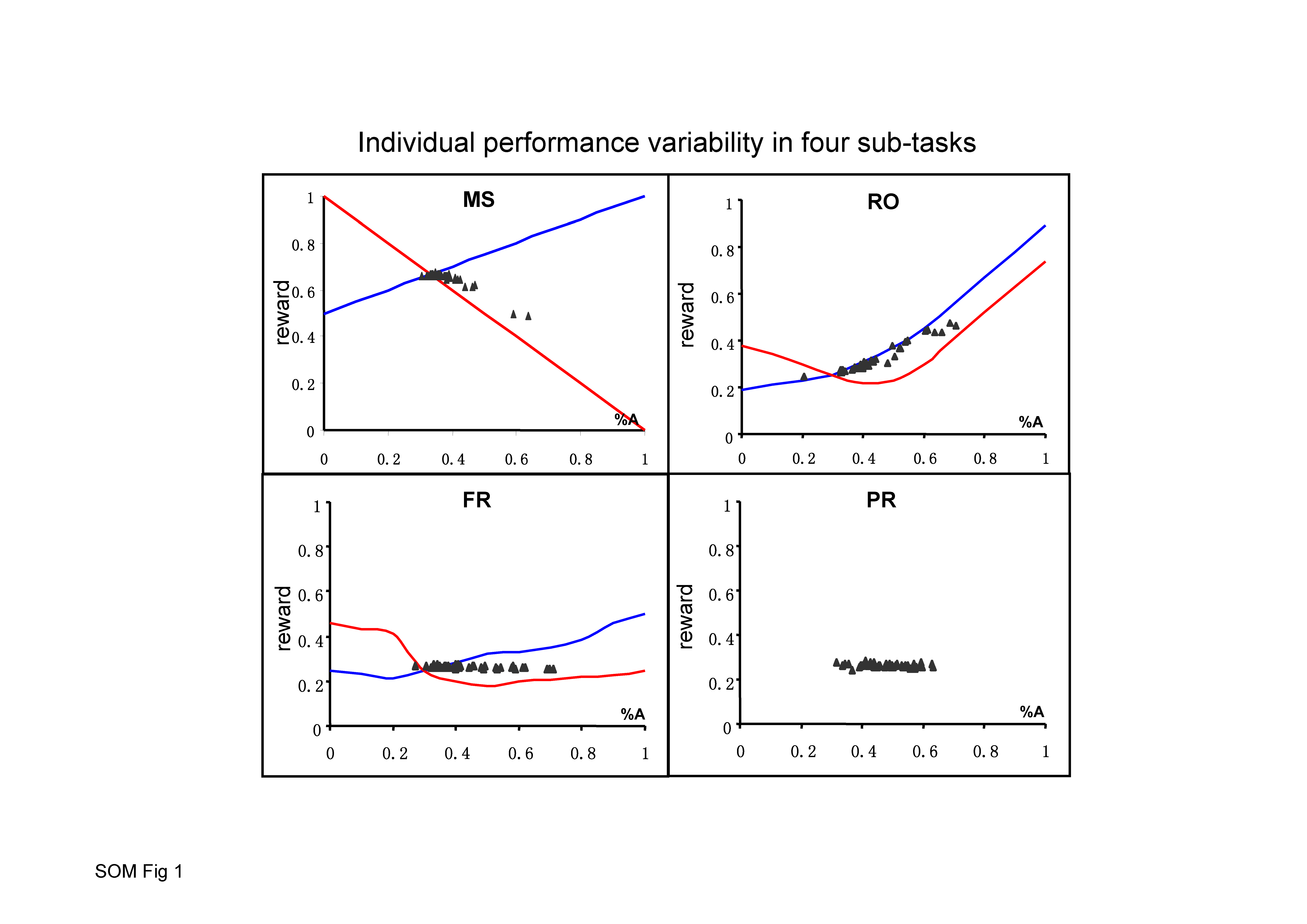

Supplement: Figure S1 — Individual subject performance variability in both tasks. These four panels represent how individual subject averagely perform in 4 different reward structures (MS, RO, FR, PR). Immediate reward subject receive from each choice they make depends on two variables: 1) current decision (A or B, Red and Blue trace correspondingly) and 2) the percentage of choice A (%A) made over the past 20 trials (x-axis). Each subject's average behavior is represented by a triangle on each reward structure plot. Most subject perform around the optimal strategy (cross point of red and blue curve) in the MS task, while in RO task, subjects tend to split along the %A and many subject were restricted to the crossing point which is not the optimal strategy anymore. In FR task, subjects were still slightly attracted by the crossing point while in the PR task subjects were randomly distributed around the 50% %A point. (1.44 MB TIF) [file pone.0000103.s001.tif]

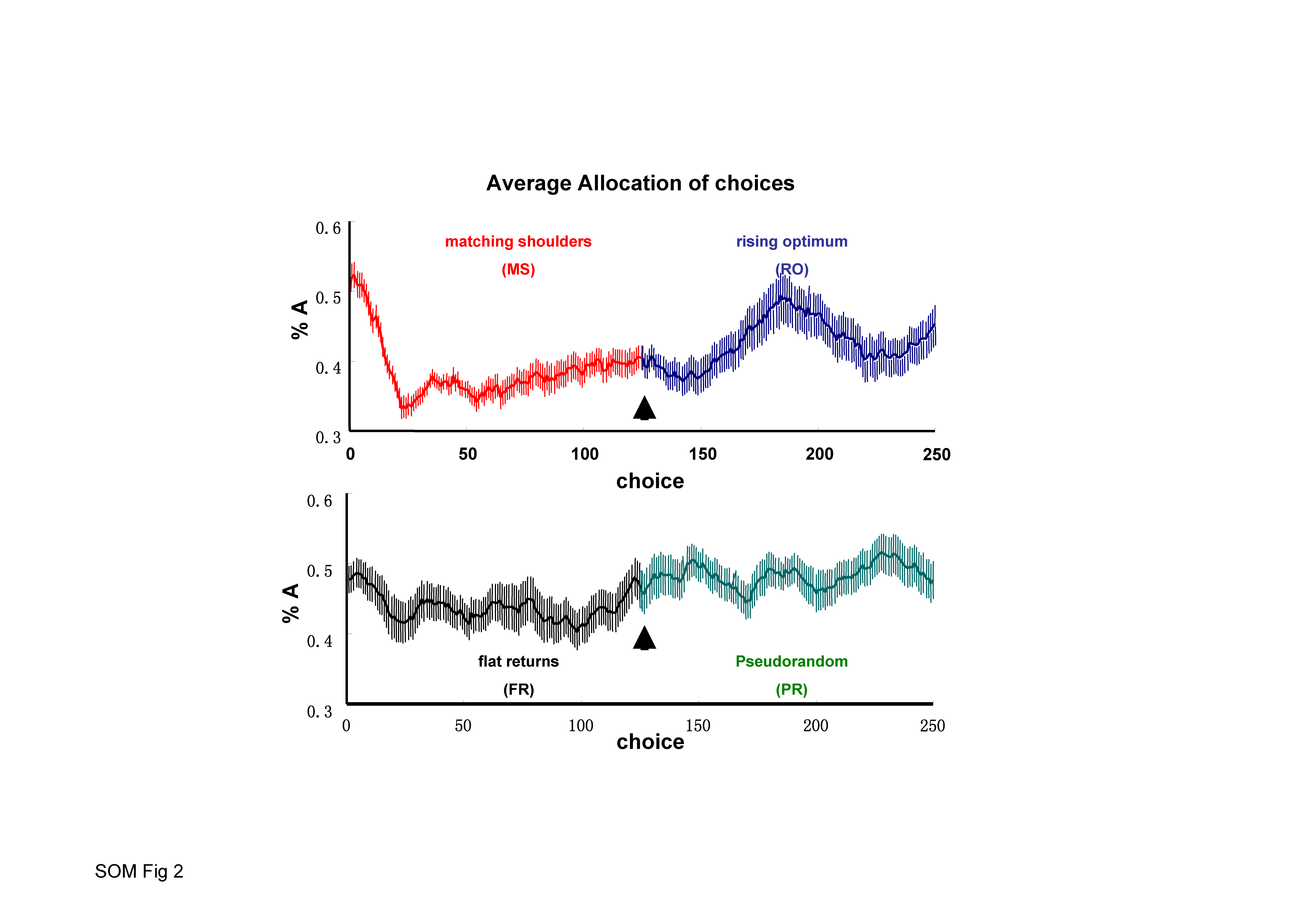

Supplement: Figure S2 — Various behavioral responses subjects performed in both tasks. Subjects quickly adjusted to the optimal strategy at the beginning of both tasks (MS and FR). The switch from matching shoulders (MS) to rising optimum (RO) reward structures was signaled by a large decrease in immediate reward return (Fig. 2) and could possibly trigger the more exploratory behavior in the RO task. However, the switch from the flat returns (FR) structure to the pseudorandom (PR) condition did not elicit a similar change in experienced reward and thus although the general behavioral patterns in FR and PR task are different (∼40% %A in FR task and ∼%50 %A in PR task), there is no evidence indicating a reliable exploratory phase in PR task. %A S.E. is indicated by vertical bars at each choice. (1.60 MB TIF) [file pone.0000103.s002.tif]

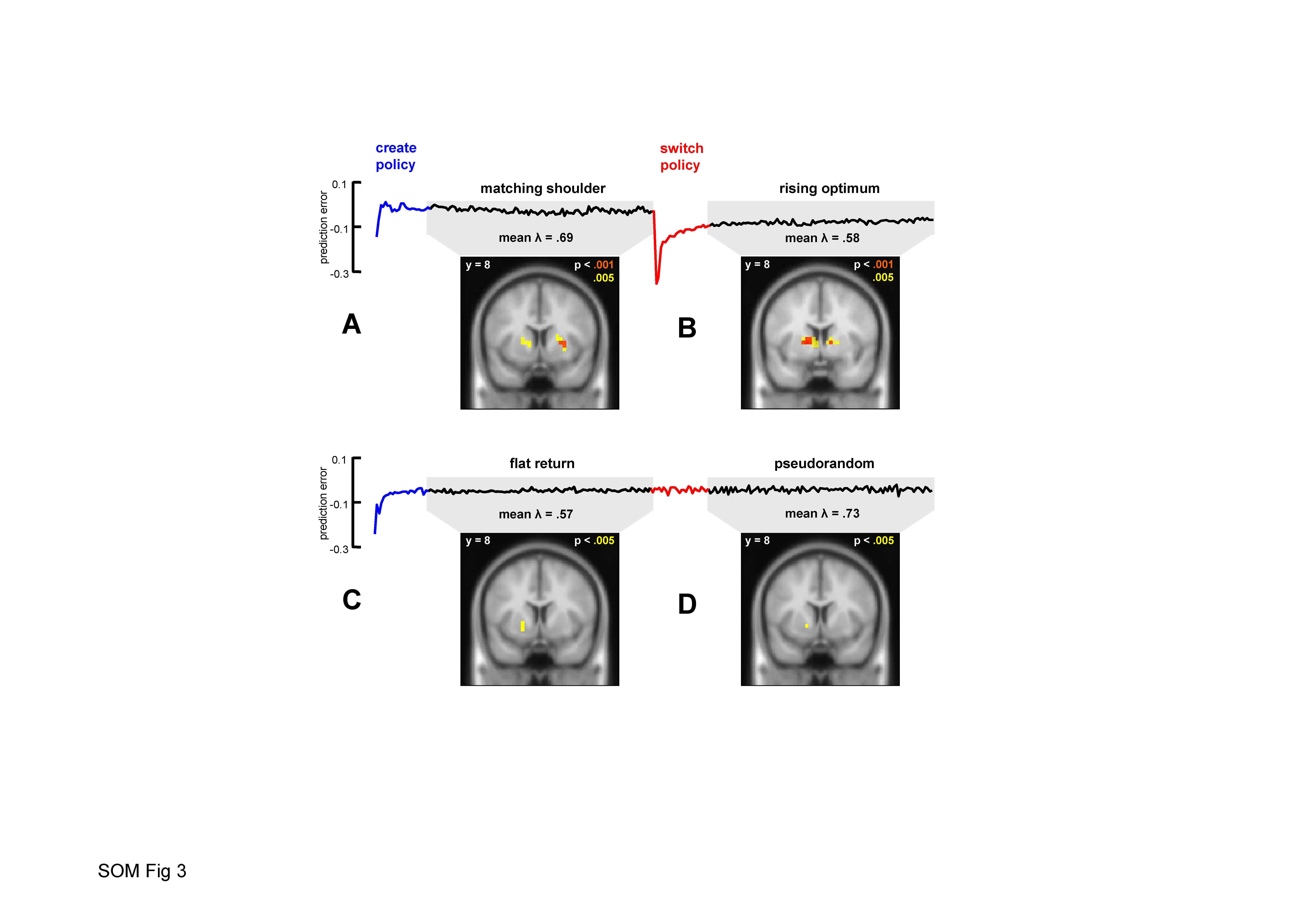

Supplement: Figure S3 — Ventral striatum as the neural correlate of average prediction error, δ(t), across subjects for four different sub-tasks using softmax method. Neural activity corresponding to prediction errors generated from independently fitting the softmax reinforcement model to 4 sub-tasks (MS, RO, FR, PR). Activity in ventral striatum correlates with the magnitude of prediction error in each sub-task (MS, RO, FR, PR) (red: p<0.001; yellow: p<0.005, uncorrected) when reward contingencies vary slowly through time. (3.82 MB TIF) [file pone.0000103.s003.tif]
